# Developmental origin of chronic diseases: toxicological implication

**DOI:** 10.2478/v10102-010-0029-8

**Published:** 2010-11

**Authors:** Štefan Bezek, Eduard Ujházy, Mojmír Mach, Jana Navarová, Michal Dubovický

**Affiliations:** Institute of Experimental Pharmacology, Slovak Academy of Sciences, 841 04 Bratislava, Slovakia

**Keywords:** developmental programming, chronic diseases, diabetes, epigenetics, prenatal and postnatal development

## Abstract

Human epidemiological and experimental animal studies show that suboptimal environments in fetal and neonatal life exerts a profound influence on physiological function and risk of disease in adult life. The molecular, cellular, metabolic, endocrine and physiological adaptations to intrauterine nutritional conditions result in permanent alterations of cellular proliferation and differentiation of tissues and organ systems, which in turn can manifest by pathological consequences or increased vulnerability to chronic diseases in adulthood. Intrauterine growth restriction (IUGR) due to intrauterine development derangements is considered the important factor in development of such diseases as essential hypertension, diabetes mellitus, ischemic diseases of the heart, osteoporosis, respiratory, neuropsychiatric and immune system diseases.

An early life exposures to dietary and environmental exposures can have a important effect on epigenetic code, resulting in diseases developed later in life. The concept of the "developmental programming" and Developmental Origins of Adult Diseases (DOHaD) has become well accepted because of the compelling animal studies that have precisely defined the outcomes of specific exposures.

The environmental pollullutants and other chemical toxicants may influence crucial cellular functions during critical periods of fetal development and permanently alter the structure or function of specific organ systems. Developmental epigenetics is believed to establish "adaptive" phenotypes to meet the demands of the later-life environment. Resulting phenotypes that match predicted later-life demands will promote health, while a high degree of mismatch will impede adaptability to later-life challenges and elevate disease risk. The rapid introduction of synthetic chemicals, environmental pollutants and medical interventions, may result in conflict with the programmed adaptive changes made during early development, and explain the alarming increases in some diseases.

## Introduction

Chronic diseases (CDs) are the greatest public health problem, either in terms of direct cost to society and government, or in terms of disability lasting for years. They include cardiovascular diseases, diabetes, cancer, osteoporosis, obesity, etc. The burden of chronic diseases is rapidly increasing worldwide. It has been calculated that in 2001 chronic diseases contributed approximately by 60% to the 56.5 million total reported deaths in the world and approximately 46% to the global burden of disease. The proportion of the burden of CDs is expected to increase to 57% by 2020. Almost half of the total chronic disease deaths are attributable to cardiovascular diseases; obesity and diabetes are also showing worrying trends, not only because they already affect a large proportion of the population but also because they have started to appear earlier in life. The origin of CDs is considered to be related to four relevant factors in fetal life are: (1) intrauterine growth retardation (IUGR); (2) premature delivery of a normal growth for gestational age fetus; (3) overnutrition in utero and (4) intergenerational factors. There is considerable evidence that IUGR is associated with an increased risk of coronary heart disease, stroke, diabetes and raised blood pressure (WHO/FAO Expert Consultation)

## Fetal Environment

The fetal environment is determined by the maternal environment and by maternal and placental physiology. Growth (an increase in the number and size of cells or in the mass of tissues) and development (changes in the structure and function of cells or tissues) of the fetus are complex biological events influenced by genetic, epigenetic, maternal maturity, as well as environmental and other factors (Wu *et al*., 2006). These factors affect the size and functional capacity of the placenta, uteroplacental transfer of nutrients and oxygen from mother to fetus, conceptus nutrient availability, fetal endocrine milieu, and metabolic pathways. Optimal fetal growth is essential for perinatal survival and has long-term consequences extending into adulthood. In conditions of severe intrauterine deprivation, there is a capacity to lose structural units such as nephrons, cardiomyocytes, or pancreatic beta-cells within developing organ systems. It is not clear if such responses are either adaptive or predictive, although it is obvious that they will result in the programming of a reduced functional capacity for life. Programming is defined as a permanent or long-term change in the physiology, morphology, or metabolism of a fetus in response to a specific insult or stimulus at a critical period in development. Any programming of an organism or tissue may be regarded as the consequence of an adaptation that is necessary to survive an insult (Barker, 1998). Developmental epigenetics is believed to establish adaptive phenotypes to meet the demands of the later-life environment. Resulting phenotypes that match predicted later-life demands will promote health, while a high degree of mismatch will impede adaptability to later-life challenges and elevate disease risk (Gluckman and Hanson, 2007).

## Epigenetic Reprogramming

Epigenetics is defined as heritable changes in gene expression that do not alter DNA sequence but are mitotically and transgenerationally inheritable. Epigenetic reprogramming is the process by which an organ genotype interacts with the environment to produce its phenotype and provides a frame-work for explaining individual variations and the uniqueness of cells, tissue, or organs despite identical genetic information. The main epigenetic mediators are histon modification DNA methylation, and non-coding RNAs. They regulate crucial cellular functions such as genome stability, gene imprinting, and reprogramming of non-imprinting genes (Tang and Ho, 2007)

**Figure 1:**
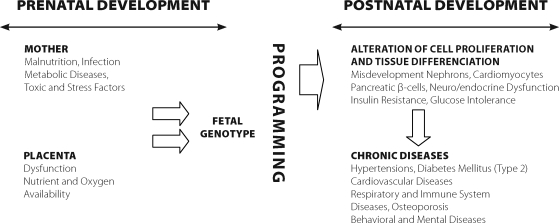
Proposed pattern of epigenetic programming of chronic diseases.

## Toxicants During Development

The periods of embryonic, fetal and infant development are remarkably susceptible to environmental hazards. The environmental pollutants and other chemical toxicants may influence crucial cellular functions during critical periods of fetal development and permanently alter the structure or function of specific organ systems. Toxic exposures to chemical pollutants during these windows of increased susceptibility can cause developmental changes, postnatal disease and disability in infants, children and across the entire span of human life. However, even subtle changes caused by chemical exposures during early development may lead to important functional deficits and increased risks of disease later in life. The timing of exposure during early development is a crucial factor to be considered in toxicological assessments (Grandjean *et al*., 2008). Emerging evidence shows that redox-sensitive signal transduction pathways are critical for developmental processes, including proliferation, differentiation, and apoptosis. As a consequence, teratogens that induce oxidative stress may induce teratogenesis via the misregulation of these pathways. Oxidizing and reducing equivalent imbalance in turn leads to macromolecule damage, namely protein modification, lipid peroxidation, and DNA oxidation, and if unchecked, oxidative damage can lead to cell death. However, oxidative stress as a mechanism does not satisfactorily explain how it might serve as a mechanism of teratogenesis (Jason and Hansen, 2006).

Disturbances during the developmental period may result in transient or irreversible long-term effects. Birth defects associated with the common air pollutants may potentially result in increased vulnerability of the respiratory and cardiovascular systems during infancy and childhood. Intrauterine growth retardation and low birth weight have been linked to alterations of respiratory function at all stages of postnatal life and intrauterine growth retardation may lead to increased susceptibility to air pollution exposure and other environmental factors (Wang and Pinkerton 2007).

## Toxicity Assessment

A major problem in assessing the contribution of the environment to human health and diseases is the small number of toxicity data in relation with the high-production volume chemicals introduced into the environment. The toxicity studies that have been performed are, in most cases, less than satisfactory for assessing the risk to human health. The existing test systems are too costly and time-consuming to evaluate more than only a small fraction of the chemicals which would need toxicity assessment. Even worse it is with very persistent and bioaccumulative substances presenting serious concerns even though there may not be evidence of toxicity. Accumulative substances may cause subtle long-term effects which may become evident in the future.

## Concluding Remarks

Finally, good biomarkers of early molecular alterations that lead to toxicity and chronic disease, resulting from environmental exposures, do not exist. Only early detection of interactions involving genes, gene-products and environmental factors could lead to the development of effective prevention strategies and elucidation of mechanisms of pathogenesis (Olden 2004).
